# Evolutionary history of the DNA repair protein, Ku, in eukaryotes and prokaryotes

**DOI:** 10.1371/journal.pone.0308593

**Published:** 2025-03-25

**Authors:** Sadikshya Rijal, Ashmita Mainali, Sandesh Acharya, Hitesh Kumar Bhattarai

**Affiliations:** Department of Biotechnology, Kathmandu University, Dhulikhel, Nepal.; Chang Gung University, TAIWAN

## Abstract

Ku is essential in non-homologous end-joining (NHEJ) across prokaryotes and eukaryotes, primarily in double-stranded breaks (DSBs) repair. It often presents as a multi-domain protein in eukaryotes, unlike their prokaryotic single-domain homologs. We systematically searched for Ku proteins across different domains of life. To elucidate the evolutionary history of the Ku protein, we constructed a maximum likelihood phylogenetic tree using Ku protein sequences from 100 representative eukaryotic, prokaryotic, and viral species. The resulting tree revealed a common node for eukaryotic Ku proteins, while viral and prokaryotic species clustered into a distinct clade. Our phylogenetic analysis reveals that the common ancestry of Ku70 and Ku80 likely resulted from a gene duplication event in the ancestral eukaryote. This inference is supported by BLASTp results, which indicate a close resemblance between archaeal Ku and eukaryotic Ku, particularly Ku70. The presence of both Ku protein paralogs in the Discoba group further supports the hypothesis that the gene duplication occurred early in eukaryotic evolution. It is plausible that archaea, which may have acted as intermediaries for Ku transfer, subsequently lost the Ku protein. Nonetheless, the extensive horizontal transfer of Ku among prokaryotes and its relatively higher prevalence in bacteria complicates our understanding of how Ku protein was inherited by early-branching eukaryotes.

## Introduction

Double-stranded breaks (DSBs) are one of the most mutagenic forms of DNA damage, which result in genome instability and potential cell death if not repaired. Cells produce DSBs either by pathologic means, such as chromosomal translocation, physical activity, reactive oxygen species, ionizing radiation, and unusual action of the nuclear enzyme on DNA, or by physiological processes like [VDJ] recombination. To treat such lethal DNA breaks, eukaryotes employ two primary mechanisms to repair DSBs: the homologous recombination (HR) pathway and the non-homologous end joining (NHEJ) pathway. HR pathway is more prevalent in early-branching eukaryotes like yeast, whereas the NHEJ pathway is predominantly used by late-branching eukaryotes [1]. HR is a more precise mechanism for repairing DSBs because it relies on extensive sequence homology to accurately restore the damaged DNA. In contrast, NHEJ is prone to errors, as it requires little to no homology, often resulting in insertions or deletions at the repair site [2]. However, NHEJ is more suited for restoring DSBs because its repair enzymes work independent of the DNA sequence order and can process various types of DSBs substrates: blunt ends, 5′ and 3′ overhangs, and DNA hairpins. This versatility enables NHEJ to restore a wide range of DNA damages in an efficient manner [3].

### The repair of DSBs in eukaryotes via the Classical NHEJ pathway (cNHEJ)

The cNHEJ pathway relies on DNA-dependent protein kinase (DNA-PK) complex that comprises of Ku and DNA-PK catalytic subunit (DNA-PKc). In the complex, the primary function of Ku is the recognition of DSBs and the recruitment of DNA-PKcs. DNA-PKcs then facilitates the repair of DSBs by phosphorylating proteins bound to the DNA, utilizing its kinase activity, and recruiting additional proteins, such as DNA ligase, that are essential for completing the repair process. In eukaryotes, the significance of Ku in cNHEJ pathway is evident from the detrimental effects reported in Ku-deficient cells, such as erroneous end-joining, radiosensitivity, chromosomal breakage, translocations, and aneuploidy [4].

### Ku structure in eukaryotes

In eukaryotes, Ku protein functions as a stable heterodimer composed of two subunits, Ku70 (70kDa) and Ku80 (80kDa). Ku proteins require the heterodimerization of both subunits [5] each of which consists of three distinct domains: an N-terminal alpha-helix/beta-barrel von Willebrand A (vWA) domain, a central beta-barrel or core domain, and a helical C-terminal domain [5]. A crystal structure of Ku70/80 reveals the dimerization of the two subunits where the central beta-barrel domains of the subunits form contact [6]. Moreover, this core domain is essential for forming the ring structure to hold the DNA duplex during the repair [7,8]. Similarly, the helical C-terminal domain (CTD) is present in both Ku subunits. The Ku80 CTD is approximately 15kDa, containing a helical and disordered region that recruits DNA-PKCs to DNA in vertebrates. This domain is missing in other eukaryotes like *Saccharomyces* and *Arabidopsis* [9,10]. In contrast, the Ku70 CTD comprises a highly flexible linker region followed by a structured 5 kDa helix-loop-helix region called the SAP domain [11]. The SAP domain seemingly has DNA binding properties as it has been demonstrated to increase the overall DNA binding affinity of the heterodimers [12,13]. The recent experimental findings in *Arabidopsis thaliana* highlight the role of SAP domain in either establishing or stabilizing the Ku protein and DNA interaction during the initial binding [14]. The C-terminal domain is also susceptible to post-translational modifications to control Ku interaction with pro-apoptotic proteins and direct homeodomain proteins to DNA ends [15,16]. Moreover, the C-terminus has been demonstrated to play an integral role in recruiting the ligase LigD to the Ku complex during NHEJ in bacteria [17]. Finally, while the function of the N-terminal vWA domain has not been fully understood, it has been demonstrated to interact with NHEJ factors during the DSB repair. This domain is also considered to be relevant for telomere control, given its association with the components of the telomere complex [7,8].

### Ku in prokaryotes

Ku proteins are absent in many prokaryotes, including the widely studied bacterial strain *Escherichia coli K12*[1,18]. The initial evidence for the presence of bacterial NHEJ was derived from *in silico* analyses, which led to the identification of homologs of Ku70/80 and ATP-dependent DNA ligase in various bacterial genomes [18,19]. Weller et al. experimentally validated bacterial cNHEJ and demonstrated that the *Mycobacterium tuberculosis* LigD (LigDMtb) protein is an ATP-dependent DNA ligase. Their findings suggest that LigDMtb is activated by its cognate KuMtb partner, likely through a direct protein-protein interaction. [20]. Following this discovery, the cNHEJ apparatus was discovered in many bacterial species, including *Bacillus subtilis, Mycobacterium tuberculosis, Streptomyces* and *Sinorhizobium meliloti*.

### Ku structure in Prokaryotes

Unlike their much larger eukaryotic counterparts (70–80 kDa), the prokaryotic Ku proteins are smaller (30–40 kDa). The conserved “Ku domain” in prokaryotes forms the core of eukaryotic Ku complexes. The bacterial Ku complexes are mostly homodimers and bind to the ends of the duplex DNA. Bacterial NHEJ comprises of Ku which recruits ATP-dependent DNA ligases to create an early DNA repair core complex [1,18]. Deactivated Ku and ligases in *Bacillus subtilis* and *Mycobacterium smegmatis* were observed to make the strains more sensitive to ionizing radiation in stationary phase and spores [20,21].

The discovery of Ku protein in eukaryotes preceded their identification in prokaryotes and archaea, underscoring its significance in eukaryotic biology. This contrasts with most DNA repair pathways, initially observed in prokaryotes, and later identified in eukaryotes. Unusually, the identification of Ku in prokaryotes and archaea proved challenging due to the less apparent conservation of Ku when comparing DNA or protein sequences across diverse phylogenetic domains. Ku in prokaryotes and archaea were identified using PSI–BLAST to the second or third iteration where the eukaryotic Ku was used as the reference [18,19]. Despite this loose sequence homology, the eukaryotic and prokaryotic counterparts share similar secondary and tertiary structures. This suggests the potential common origin of bacterial Ku, Ku70, and Ku80 proteins [18,19].

This paper undertakes a thorough exploration to identify Ku proteins across viral, bacterial, archaeal, and eukaryotic species. The study of inheritance patterns of Ku among prokaryotes has been challenging due to the extensive horizontal transfer of genes [22]. In this study, we investigated the evolutionary trend of Ku across different domains of life using phylogenetic analysis. Additionally, the study delves into domain architecture analysis to study the anticipated paralogous relationship between Ku70 and Ku80 proteins.

## Results

### Distribution of Ku across prokaryotes, eukaryotes and viruses

To study the evolution of the Ku protein in bacteria, we selected 122 bacterial species spanning most phyla in the bacterial domain. 272 amino acids long Ku protein from *Mycobacterium tuberculosis* [UniProt Accession ID: P9WNV3] was used as a query to search for homologs in these species. The blastp of *M. tuberculosis* Ku protein against the selected bacterial species resulted in positive hits in only 30 bacterial species (Table 1a). According to the orthologous protein database (OrthoDB) Actinomycetota, Proteobacteria, and Firmicutes are the top three bacterial classes which dominantly possess the Ku protein homolog. The database records the presence of prokaryotic Ku protein in about one-third of bacterial species, showing that Ku is unevenly present among the bacteria. On the other hand, only 19 archaeal Ku protein sequences were featured in the OrthoDB database suggesting that only a few archaeal species harbor Ku protein (Table 1b). Likewise, OrthoDB featured 11 Ku protein sequences in the virus (Table 2).

**Table 1:** List of prokaryotic representative species in Fig 1.

Actinomycetota	Alphaproteobacteria	Chlamidiota
*Nocardia brasiliensis AFU03477.1*	*Rhizobium leguminosarum CAK07635.1*	*Parachlamydia acanthamoebae CCB86018.1*
*Rhodococcus jostii ABG98966.1*	*Bradyrhizobium japonicum BAL13505.1 | 1 | *	**Thermodesulfobacteriota**
*Streptomyces sviceus EDY54145.1*	*Bradyrhizobium japonicum BAL07271.1 | 2 | *	*Desulfomonile tiedjei AFM25046.1*
*Saccharothrix espanaensis CCH31573.1 | 1 | *	*Bradyrhizobium japonicum BAL07922.1 | 3 | *	*Thermodesulfatator indicus AEH45291.1*
*Saccharothrix espanaensis CCH315712.1 | 2 | *	**Bacteriodota**	**Betaproteobacteria**
*Streptomyces ambofaciens WP053142817.1 | 1 | *	*Niastella koreensis AEV97944.1 | 1 | *	*Cupriavidus necator AEI81269.1 | 1 | *
*Streptomyces ambofaciens WP053141159.1 | 2 | *	*Niastella koreensis AEW00550.1 | 2 | *	*Cupriavidus necator AEI812225.1 | P | *
*Streptomyces ambofaciens WP053141182.1 | 3 | *	*Chitinophaga pinensis ACU58495.1*	*Paraburkholderia xenovorans ABE30641.1*
*Streptantibioticu_cattleyicolor AEW97828.1*	**Acidobacteriota**	*Achromobacter xylosoxidans ADP13795.1*
*Streptomyces coelicolor AAF23071.2*	*Candidatus Solibacter | AB | 88738.1*	*Delftia acidovorans ABX36971.1*
*Streptomyces griseus WP030705942.1 | 1 | *	*Terriglobus roseus AFL88371.1*	**Deltaproteobacteria**
*Streptomyces griseus WP164364672.1 | 2 | *	*Acidobacterium capsulatum C1F735.1*	*Haliangium ochraceum ACY15831.1*
*Streptomyces griseus WP115068043.1 | 3 | *	*Granulicella mallensis AEU35712.1*	**Firmicutes**
*Streptomyces avermitilis BAC70656.1 | 1 | *	**Verrucomicrobiota**	*Paenibacillus mucilaginosus AFH65824.1*
*Streptomyces avermitilis Q82PM3.1 | 2 | *	*Opitutus terrae B1ZWL2.1*	*Bacillus thuringiensis AFU11505.1*
*Streptomyces noursei WP_157955381.1 | 2 | *		
*Streptomyces noursei WP_044379509.1 | 3 | *		
*Streptomyces noursei WP_064072575.1 | 4 | *		
**Euryarchaeota**	**Thaumarchaeota**	
*Geoglobus ahangari*	*Nitrososphaera sp. AFS*	
*Methanolinea mesophila*	*Candidatus Nitrosocosmicus exaquare*	
*Methanobacterium formicicum DSM3637*	*Candidatus Nitrosocosmicus arcticus*	
*Candidatus Methanoperedens sp. BLZ2*	*Candidatus Nitrosocosmicus oleophilus*	
*Archaeoglobus fulgidus DSM4304*		
*Methanobacterium lacus*		
*Methanobacterium formicicum*		
*Archaeoglobus veneficus SNP6*		
*Methanobacterium aggregans*		
*Methanobacterium paludis*		
*Methanothrix soehngenii GP6*		
*Archaeoglobus sulfaticallidus PM70-1*		
*Methanobacterium sp. MethCAN*		
*Methanobacterium sp. SMA-27*		
*Methanocella paludicola SANAE*		

(a) These bacterial Ku protein sequences were obtained by blasting *M. tuberculosis* Ku protein sequence as input query against 122 prokaryotes from different phyla [23]. (b) 19 archaeal species consisting of Ku70/80 beta-barrel domain obtained from OrthoDB. Their ortholog identifier number is ‘147616at2157’.

**Table 2:** 11 viral sequences consisting of Ku70/80 beta-barrel domain.

Virus	
*Mycobacterium_virus_Omega*	*Mycobacterium_phage_Ariel*
*Mycobacterium_virus_Baka*	*Mycobacterium_phage_Redno2*
*Mycobacterium_virus_Littlee*	*Mycobacterium_phage_Minerva*
*Mycobacterium_virus_Corndog*	*Mycobacterium_phage_Thibault*
*Streptomyces_phage_BillNye*	*Mycobacterium_phage_MiaZeal*
*Mycobacterium_phage_Wanda*	

Viral sequences retrieved from OrthoDB were grouped under the ortholog identifier number ‘5222at10239’ in the database.

OrthoDB search for Ku70 and Ku80 in eukaryotes indicates a high prevalence in Animalia, Fungi, and Chloroplastida. The eight eukaryotic groups included in this study are Discoba, Metamonada, Amoebozoa, Chloroplastida, Glaucophyte, Rhodophyta, Alveolata, and Opisthokonta [24]. Opisthokonts are further classified as ‘Animalia’, ‘Fungi’, ‘Choanoflagellate’, and ‘Ichthyosporea’ for better resolution in the phylogenetic analysis. The organisms are divided into three groups: groups with both Ku70 and Ku80, groups without Ku70 and Ku80, and groups with Ku70 but without Ku80 (Table 3). All the species that harbored Ku80 also had Ku70 protein. 35 out of 61 eukaryotes featured both Ku70 and Ku80 proteins. Among the early-branching eukaryotes such as Hemimastigophora, Malawimonadida, Metamonada, and Discoba, Ku protein presence was only detected in Discoba. As the exact branching order of these groups is unclear, it is difficult to determine whether the Ku gene was lost from some of these groups or vertically transferred to an ancestor of Discoba and other eukaryotes. It is also possible that we did not detect Ku in these early-branching groups due to the lack of genome data. We observed that Ku70 and Ku80 were prevalent in almost all species of animals and fungi. Some unicellular parasites from Discoba and Amoebozoa harbored only Ku70 protein. These parasitic eukaryotes might have always had Ku70 only, or the potential absence of Ku80 might have been due to the loss of the Ku80 from their genomes since conventional NHEJ is dispensable in these groups of organisms [25]. For the remaining phylum, Ku protein was discovered in some species but not detected in other species. One interesting finding is that although protein blast doesn’t detect the presence of Ku protein in *Moniliophthora perniciosa,* this protein was detected in UniProt (ID: E2LAE6). However, InterPro (ID: IPR005161) records the presence of only the N-terminal domain of *M. perniciosa* Ku protein. This might be due to incomplete sequencing, incomplete annotation, or the loss of the remainder of the Ku protein domain in this organism. Overall, Ku protein was observed in early eukaryotes, with a more pronounced prevalence in higher eukaryotes. However, it’s worth noting that certain species do not report the presence of Ku at all.

**Table 3:** List of 61 representative eukaryotes in which the presence of Ku70/80 was searched in OrthodB.

	Presence of Ku70 and Ku80	Presence of only Ku70	Absence of both
**Animalia**	*Strongylocentrotus purpuratus*		*Buddenbrokia plumatellae*
	*Nematostella vectensis*		
	*Hydra vulgaris*		
	*Monodelphus domestica*		
	*Homo sapiens*		
	*Branchiostoma floridae*		
**Choanoflagellate**	*Monosiga brevicollis*		
**Fungi**	*Ustilago maydis*		*Moniliophthora perniciosa*
	*Lacaria bicolori*		
	*Puccinia graminis*		
	*Schizosaccharomyces pombe*		
	*Aspergillus oryzae*		
	*Talaromyces marneffei*		
	*Saccharomyces cerevisiae*		
	*Malassezia globosa*		
	*Neurospora crassa*		
	*Candida albicans*		
	*Mortierella alpina*		
**Ichthyosporea**	*Sphaeroforma arctica*		
**Chloroplastida**	*Arabidopsis thaliana*		*Scenedesmus obliquus*
	*Volvox carteri*		*Acetabularia acetabulum*
	*Ostreococcus lucimarinus*		*Micromonas*
	*Chlamydomonas reinhardtii*		
	*Zea Mays*		
**Discoba**	*Trypanosoma cruzi*	*Leishmania aethiopica*	*Euglena gracilis*
	*Trypanosoma brucei*		*Sawyeria marylandensis*
	*Leishmania infantum*		*Reclinomonas americana*
	*Leishmania major*		*Diplonema papillatum*
	*Leishmania donovani*		
**Amoebozoa**	*Acanthamoeba castellanii*	*Entamoeba invadens*	*Physarum polycephalum*
	*Dictyostelium discoideum*	*Entamoeba histolytica*	*Hyperamoeba dachnya*
			*Mastigamoeba balamuthi*
**Alveolata**	*Oxytricha trifallax*		*Euplotes octocarinatus*
	*Stylonychia lemnae*		*Euplotes crassus*
	*Tetrahymena thermophila*		*Anophryoides haemophila*
	*Paramecium tetraurelia*		
**Glaucophyta**			*Cyanophora paradoxa*
			*Glaucocystis nostochinearum*
**Rhodophyta**			*Cyanidioschyzon merolae*
			*Griffithsia japonica*
**Metamonada**			*Spironucleus vortens*
			*Streblomastix strix*
			*Giardia lamblia*
			*Trichomonas vaginalis*

The eukaryotic species are divided into: ‘Presence of both Ku70 and Ku80’, ‘Presence of Ku70 only’, and ‘Absence of both Ku70 and Ku80’. Some unicellular parasites from Discoba and Amoebozoa were found to contain Ku70 only while the Ku protein was completely absent in the chosen Metamonada, Rhodophyta, and Glaucophyta species.

### Evolution and history of Ku proteins across three domains of life

**Fig 1:**
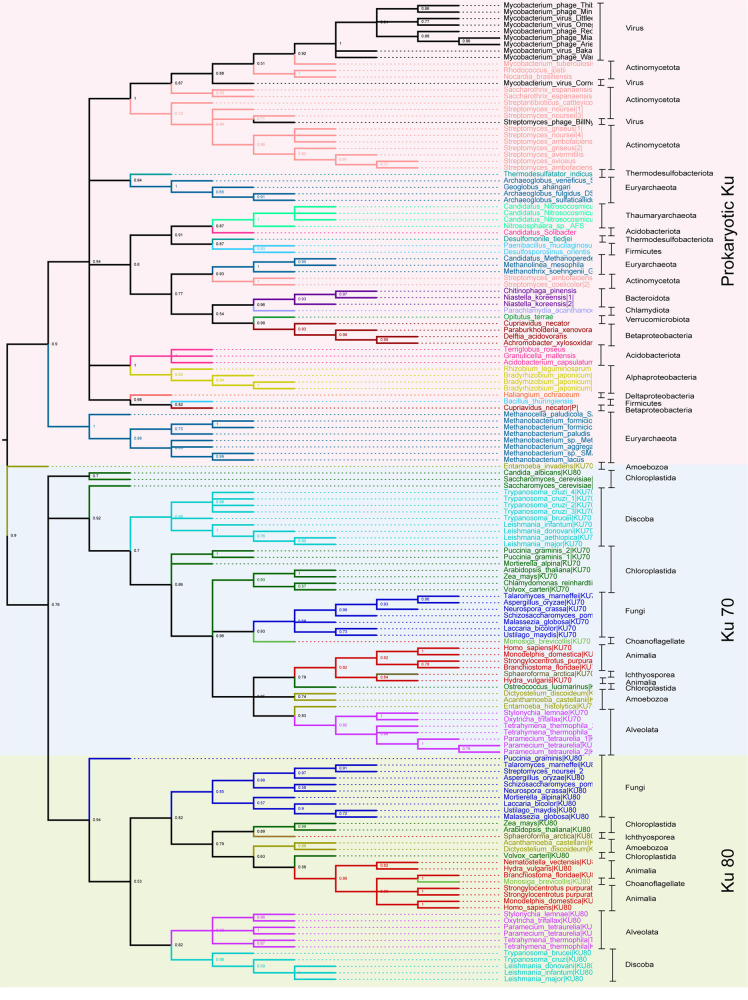
A representative Ku phylogenetic tree of 100 Ku70, Ku80, viral, and prokaryotic Ku sequences. The tree was drawn using the Maximum Likelihood method in PhyML. Automatic model selection based on the lowest BIC (Bayesian Information Criterion) was done using Smart Model Selection (SMS). Support for each branch was established using Shimodaira–Hasegawa [SH]-aLRT (approximate Likelihood Ratio Test). Mid-point rooting was performed using Mega11. Unsupported nodes ([SH]-aLRT < 50%) are excluded.

**Fig 2:**
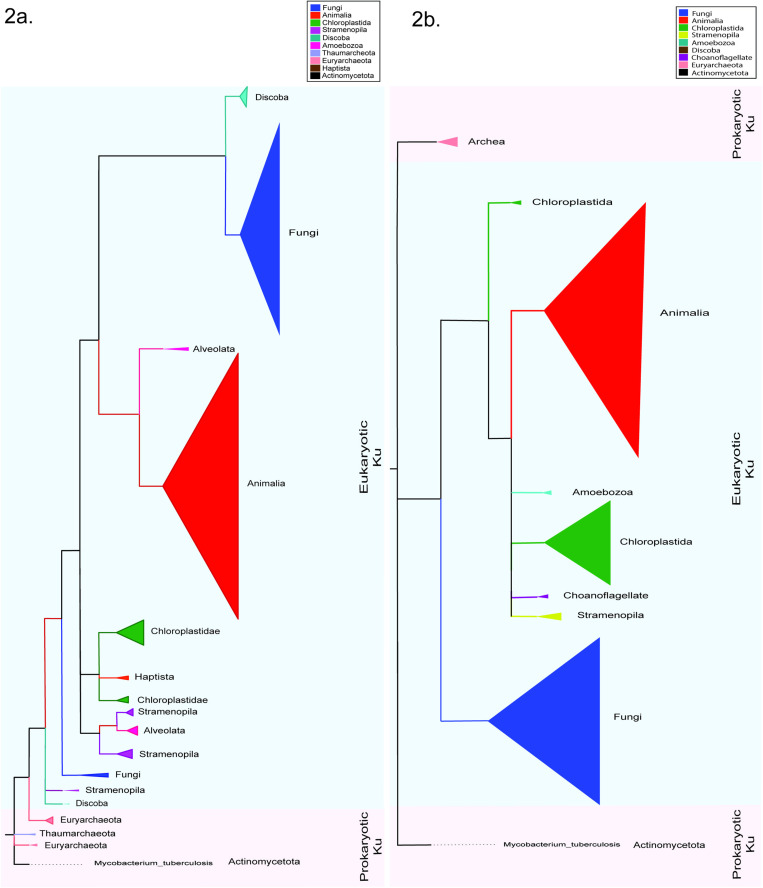
Collapsed phylogenetic trees of (a) 1097 Ku70 orthoDB sequences and (b) 1256 Ku80 OrthoDB sequences. Trees were drawn using the Maximum Likelihood method in PhyML. Automatic model selection based on the lowest BIC (Bayesian Information Criterion) was done using Smart Model Selection (SMS). Support for each branch was established using Shimodaira–Hasegawa [SH]-aLRT (approximate Likelihood Ratio Test). Trees have been simplified for better visualization using Figtree. Monophyletic clades are collapsed into triangles. The clades that harbored species spanning multiple groups were annotated based on the dominant group. The area of the triangle is not proportional to the number of Ku sequences. The trees were rooted using *M. tuberculosis* as the outgroup.

Fig 1 contains 100 selected bacterial, archaeal, eukaryotic, and viral Ku across diverse classes (Table 4). Ku proteins in *Cupriavidus necator* plasmid and *T. thermophila* transposon are also included in the tree. Fig 2a was built using Ku70/80 beta-barrel sequences of 1097 Ku70 proteins and 19 archaeal Ku sequences (S1 Fig). Likewise, Fig 2b was constructed with 1256 Ku80 proteins and 19 archaeal Ku sequences (S2 Fig). The prokaryotic Ku sequence from *M. tuberculosis* was used for outgroup rooting in Fig 2a and 2b.

**Table 4:** The number of different groups of organisms included in Fig 1.

Group/Kingdom/Phylum	Frequency in the general tree
Fungi	8
Animalia	7
Ichthyosporea	1
Chloroplastida	5
Choanoflagellate	1
Alveolata	7
Amoebozoa	4
Discoba	6
Actinomycetota	11
Firmicutes	3
Alphaproteobacteria	4
Bacteriodota	3
Thermodesulfobacteriota	2
Deltaproteobacteria	1
Chlamydiota	1
Acidobacteria	4
Verrucomicrobiota	1
Euryarchaeota	15
Thaumarchaeota	4
Virus	11

Overall, the Ku80 and Ku70 genes cluster into separate clades confirming that these paralogs duplicated early in eukaryote evolution. The only exception is Ku70 from the amoebozoan *Entamoeba invadens*, which clustered separately from all other eukaryotes. We could not conclude whether this placement is an artifact or a separate independent gene transfer from prokaryotes. NCBI blastp of the *E. invadens* resulted in unclassified Ku sequences from different *Entamoeba* species. Like *E. invadens,* the resulting sequences only featured the core Ku domain which is probably incompletely documented or erroneously labelled. Despite being within the eukaryotic Ku protein clade, *S. cerevisiae* and *C. albicans* Ku80 appear to be an outgroup of Ku80. This might be due to the lower sequence divergence between Ku70 and Ku80 in yeast. However, the yeast *Schizosaccharomyces pombe* formed clades with the ascomycetes Ku70 and Ku80, respectively. This aligns with the literature that fission yeasts diverged from ascomycetes [26]. Discoba, the earliest eukaryote in which Ku paralogs have been identified, forms its distinct clade within the Ku70 clade. However, Discoba and Alveolata form a sister clade to the rest of Ku80.

The prokaryotic Ku forms a distinct clade apart from eukaryotic Ku, suggesting a closer homology between the bacteria and the archaea, as displayed in Fig 1. The majority of Actinomycetota Ku form a distinct sub-clade with their respective viral Ku. The proximity of *Nocardia brasilensis and Rhodococcus josti* to *Mycobacterium virus Corndog* might suggest a role of horizontal gene transfers via viruses. We could observe all the Mycobacteriophages form a clade with their host, *M. tuberculosis.* Similarly, *Streptomyces phage BillNye* forms a clade with *Streptomyces* species. *Streptomyces ambofaciens* and *Streptomyces coelicolor* are clustered together in a separate sister clade with euryarchaeota, like *Methanothrix soehngenii*, *Methanolinea mesophila* and *Candidatus methanoperedens.* The presence of Firmicutes like *Paenibacillus mucilaginosus* and *Desulfosporosinus orientis*, Acidobacter like *Candidatus solibacter* and Thermodesulfobacteriota like *Desulfomonile tiedjei and Thermodesulfatator indicus* among Euryarchaeota is reported. Similarly, Alphaproteobacteria like *Rhizobium leguminosarum* and *Bradyrhizobium japonicum*, and Acidobacteria like *Granulicella mallensi*, *Acidobacterium capsulatum* and *Terriglobus roseus* are clustered together. Additionally, Bacteroidota like *Niastella koreensis* and *Chitinophaga pinensis*, Chlamydiota like *Parachlamydia acanthamoebae*, Verrucomicrobiota like *Opitutus terrae*, and Betaproteobactera like *C. necator*, *Paraburkholderia xenovorans*, *Delftia acidovorans* and *Achromobacter xylosoxidans* are clustered together. However, the plasmid Ku of *C. necator* is present separately with *Bacillus thuringiensis* and *Haliangium ochraceum, suggesting the roles of plasmids in gene transfer*. Likewise, we could further observe a *Streptomyces noursei* Ku homolog interspersed within the fungal Ku80 sub-clade, which may have been due to shared habitat.

Interestingly, *Methanobactrium* and *Methanocella* species of Euryarchaeota form a distinct sister clade to the bacterial clade, whereas other archaeal species are found interspersed. When the Euryarchaeota *Methanobacterium lacus* was subjected to a standard blastp algorithm against eukarya, 36 results were displayed: eight featuring prokaryotic Ku domains, 26 featuring eukaryotic Ku70 domains, and two with unidentified domains. In contrast, blasting *Mycobacterium tuberculosis* against eukarya resulted in all 25 hits featuring prokaryotic Ku. Similarly, standard blastp of *Archeoglobulus fulgidus Ku* protein, which lies immersed in the bacterial core clade, against eukarya, resulted in 31 species, all of which harbored prokaryotic Ku domains. This observation hints at the proximity of eukaryotic Ku70 to archaea, particularly some Euryarchaeota. More strikingly, *Methanobacterium and Methanocella* species constitute a sister clade to the eukaryotic Ku70 clade in Fig 2a. However, a distinct clade of archaea can be observed apart from the eukaryotic Ku80 clade in Fig 2b.

### Domain architecture of Ku Protein

Prokaryotic Ku, except for *Streptomyces coelicolor,* only has a central Ku core domain. The Ku protein of *S. coelicolor (SCF55.25c)* has around 40 amino acid long C-terminal Helix–Extended-region–Helix (HEH) extension [19]Multiple domain architectures have been identified in the Ku protein of eukaryotes, making its study paramount in understanding its evolutionary trend.

**Fig 3:**
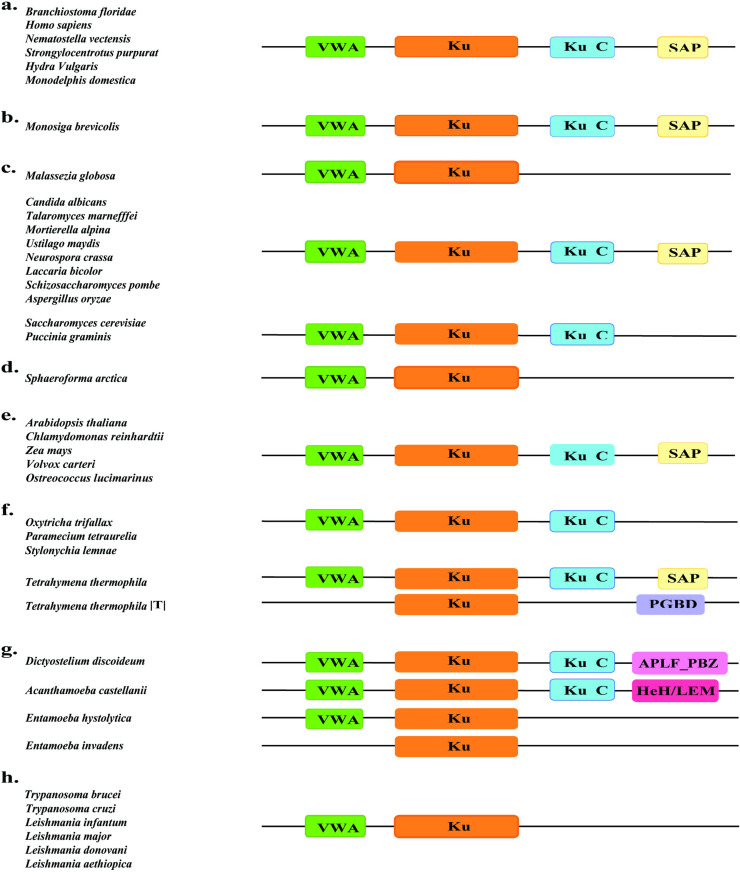
VWA, KU70/80 beta-barrel domain, Ku C terminal, and other Ku70 domains of representative species determined using InterPro. (a) Animalia (b) Choanoflagellate (c) Fungi. (d) Ichthyosporea (e) Chloroplastida (f) Alveolata (g) Amoebozoa (h) Discoba.

Analysis of the domain architecture of Ku70 from 38 representative species reveals at least seven different architectures according to InterPro, as shown in Fig 3. The central Ku beta-barrel domain is often flanked by an N-terminal vWA domain and a helical C-terminal domain (Fig 3a). At the end of the C terminus lies the SAP domain. The SAP domain is prevalent across all kingdoms in eukaryotes except Discoba, Amoebozoa, Haptophyta, and some Fungi and Alveolata [27]. Some amoebozoa, like *A. castellanii,* have HEH/LEM domain after their C-terminus (Fig 3g). Besides, InterPro search revealed one Apusomonadidae and two Opisthokonta that have HEH domain at the C-terminus of Ku70/80 beta-barrel domain. HEH domain has been evolutionarily linked to the SAP domain, where the SAP was speculated to be the eukaryotic version of the HEH domain previously identified in *S. coelicolor* [19]. Amoebozoa, like *D. discoideum, have* an Aprataxin and PNK-like factor, PBZ domain (APLF_PBZ) domain after their C-terminus (Fig 3g). APLF has been identified as a DNA damage response protein, and PBZ is a zinc finger motif widespread in eukaryotes, notably in *D. discoideum* [27]. Besides *D. discoideum,* InterPro features five more Amoebozoans that harbor the APLF_PBZ domain on their Ku70 C-terminus: *Tieghemostelium lacteum, Planoprotostelium fungivorum, Polysphondylium violaceum, Heterostelium pallidum,* and *Cavenderia fasciculata*. *E. invadens*, which are excluded from the eukaryotic Ku protein clade in Fig 1, only have the core Ku domain. Lastly, Ku70 in the transposon of *Tetrahymena thermophila* has the central core domain and a PiggyBac Domain (PGDB) (Fig 3f).

**Fig 4:**
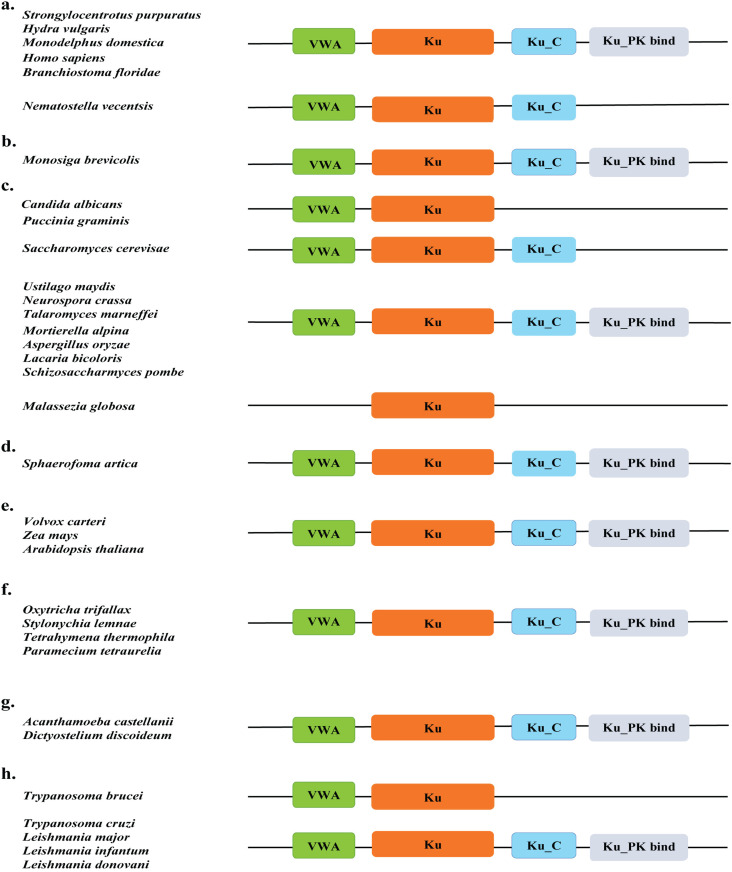
VWA, KU70/80 beta-barrel domain, Ku C terminal, and other Ku80 domains of representative species determined using InterPro. (a) Animalia (b) Choanoflagellate (c) Fungi. (d) Ichthyosporea (e) Chloroplastida (f) Alveolata (g) Amoebozoa (h) Discoba.

Similarly, an analysis of the Ku80 proteins among the 61 species reveals at least four different domain architectures, according to InterPro (Fig 4). Consistent with the literature, the central Ku beta-barrel domain is flanked by the N-terminal vWA domain and C-terminus. At the end of the C terminus often lies the DNA PK binding domains (Fig 4a). The importance of DNA-PK signaling for DNA DSBs repair has been recognized as early as in Amoebozoa *Dictyostelium discoideum* [28]. This domain architecture is conserved in all Ku80 proteins of Amoebozoa, Alveolata, and Chloroplastida (Fig 4e, 4f, and 4g). All the species from the Animalia kingdom except *Nematostella vectensis* share the conventional Ku80 domain architecture (Fig 4a). Similarly, almost all Fungi Ku80 possess the Ku-PK-bind domain (Fig 4c). It has been previously reported in organisms devoid of DNA-PKCs, Ku80 domain architecture lacks this C-terminal extension [29]. Yeast also lacks DNA-PKCs and DNA-PK binding domains in Ku80 [30]. Interestingly, while *S. cerevisiae* displays domain architecture as predicted, *Schizosaccharomyces pombe* does have a C-terminal DNA-PK binding domain. We got domain hits for Ku-PK-Bind for four out of five Discoba species (Fig 4h).

### AlphaFold models of Ku core proteins across species

The structural conservation of the Ku core domain across various species was investigated using AlphaFold models. All protein subsets, including the experimental models, exhibited an antiparallel beta-barrel structure (Fig 6, S3 Fig). RMSD values were similar between the human experimental Ku70 and Ku80 core domains and those from other species (S1 Table). The maximum RMSD value of 5.871 was observed between the human Ku70 core and *Saccharomyces cerevisiae* Ku70, while the minimum RMSD value occurred between the human Ku70 core and *Acanthamoeba castellanii* Ku70. Notably, the human Ku70 core shows consistently lower RMSD values when compared to eukaryotic Ku70 and most Ku80 proteins than to prokaryotic counterparts, a trend also observed for the human Ku80 core. Overall, our results corroborate with previously established findings, demonstrating that the core Ku domains are highly conserved across species.

**Fig 5:**
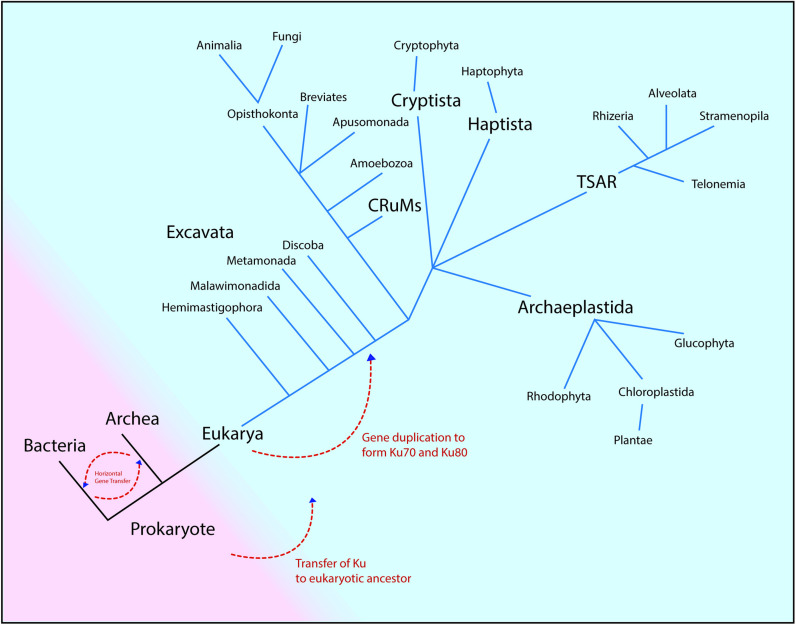
A model of inheritance of Ku across domains [21]. The figure above describes the phylogenetic classification of the domain eukarya into groups [23,31]. The earliest eukaryotes possibly inherited primitive Ku protein. Gene duplication might have occurred in some ancestral Excavate, leading to the formation of Ku70 and Ku80. These Ku proteins were then vertically inherited by eukaryotic groups: Discoba, Opisthokonta, Amoebozoa, TSAR, and Archaeplastida.

**Fig 6:**
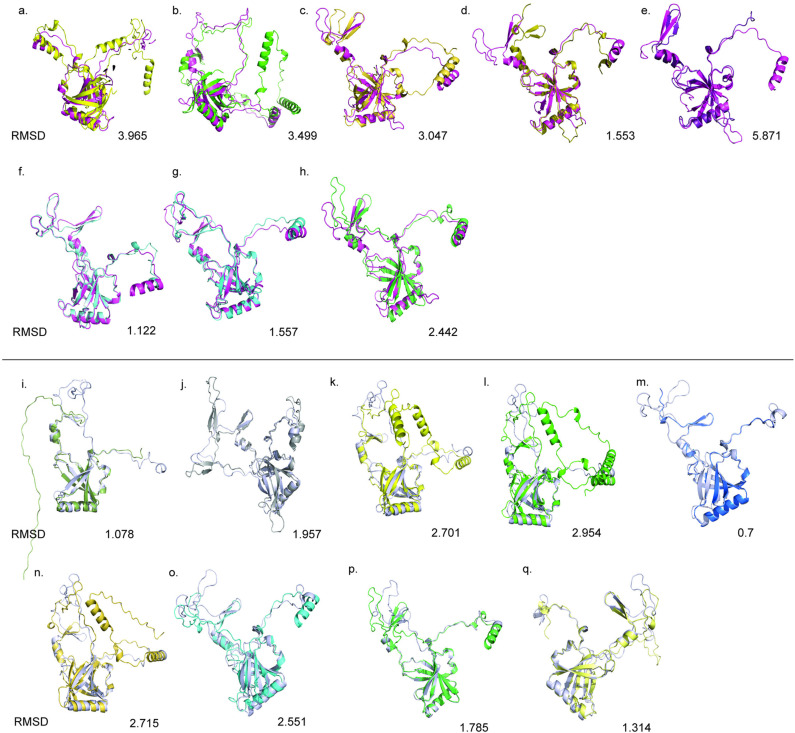
Structural Overlay of Experimental Human Ku Core Protein Against Protein Subsets from Various Species. (a-h) Overlay of the human Ku70 core (magenta) with the Ku70 core from *Mycobacterium phage Thibault*, *Mycobacterium tuberculosis*, *Methanocella paludicola SANAE*, *Trypanosoma cruzi KU70, Saccharomyces cerevisiae KU80*, *Arabidopsis thaliana Ku70*, *Homo sapiens Ku70*, and *Homo sapiens Ku80*. (i-q) Overlay of the human Ku80 core (grey) with the Ku80 core from *Trypanosoma brucei KU80, Saccharomyces cerevisiae KU80, Mycobacterium phage Thibault*, *Mycobacterium tuberculosis*, *Tetrahymena thermophila* transposon, *Methanocella paludicola SANAE, Homo sapiens Ku70, Arabidopsis thaliana Ku80*, and *Homo sapiens Ku80.*

## Discussion

### Ku in prokaryotes and Ku70 and Ku80 in eukaryotes arise from a Common Ancestor

Aravind et. al previously proposed a model based on the examination of Ku domain architecture across various domains of life, suggesting that prokaryotic Ku represents the ancestral form [19]. Our study, which includes phylogenetic analysis of a wide range of eukaryotic Ku homologs, and a comparison of the protein domain structures, supports this model. As illustrated in Fig 1, prokaryotic and eukaryotic Ku proteins trace back to a common ancestor. Bacterial Ku and eukaryotic Ku form a distinct clade, with archaeal Ku interspersed among bacterial Ku, aligning with the notion that bacterial and archaeal Ku share a primitive core domain. Throughout evolution after passing from the last common bacterial or archaeal ancestor, Ku proteins might have undergone functional diversification via gene duplication in eukaryotes, resulting in two distinct paralogs of the Ku protein.

### Different modes of Ku protein inheritance

This paper reports different modes of inheritance for the Ku protein throughout evolutionary history (Fig 5). The vertical inheritance of Ku among the same domains of life is evident in Fig 1, S1 Fig, and S2 Fig. Notably, the extensive horizontal gene transfer of Ku protein has been recorded previously [22]. Fig 1 aligns with the literature making the evolutionary scenario of Ku protein origin indistinct. Within bacteria, instances of horizontal inheritance of Ku may have been facilitated by the viral carriers. Moreover, the presence of Ku has been reported in the plasmids of Alpha-proteobacteria, Beta-proteobacteria, and Streptomycetaceae, which may have further facilitated the horizontal transfer of Ku protein among the prokaryotes [22]. In our investigation, one plasmid Ku of *C. necator* was found along with Firmicutes and Delta-proteobacteria. Ku transfers between Alphaproteobacterium(donor) and the common ancestor(recipient) of Acidobacteria, and the common ancestor of Delta-proteobacteria has been previously documented. Similarly, some alphaproteobacteria and some acidobacteria are clustered together in a sister clade in Fig 1, suggesting the role of horizontal gene transfer. Strikingly, the presence of Firmicutes and Actinobacteria like *S. ambofaciens* and *S. coelicolor* among Euryarchaeota clades hints towards a possible horizontal transfer from archaea to these classes of bacteria as the transfer of Ku from archaea to firmicutes and actinobacteria has been previously reported [22]. The presence of Ku70 and Ku80 in a distinct clade from prokaryotic Ku suggests duplication of ancestral eukaryotic Ku. Notably, the proximity of Ku70 to Euryarchaea (*Methanobacterium* & *Methanocella*), as reported from the NCBI standard blast, suggests a possible transfer of Ku proteins from archaea to eukaryotes in the early stage of inheritance or a shared ancestry.

### Domain architecture of Ku proteins

In the examination of the domain architecture of Ku70 and Ku80 proteins, our finding resonated with the earlier studies in the identification of the Ku70/80 beta-barrel as the core domain, flanked by an N-terminal von Willebrand factor A (vWA) domain and a helical C-terminal domain. While the vWA domain is conserved in Ku proteins of most eukaryotes, the C-terminal domain emerges as the most divergent region in Ku70 and Ku80 proteins, consistent with earlier observations in the literature. Notably, while the Ku80 C-terminal Ku-PK-Bind domain was initially presumed to be absent in “lower” eukaryotes, our study reveals the presence of this C-terminal extension even in early-branching eukaryotes like Discoba. Therefore, these findings suggest that the Ku-PK-Bind domain addition to the Ku80 C-terminus might have occurred in an early-branching eukaryotic ancestor. Instead, loss of this domain might have been triggered later in organisms that lack DNA-PKCs or in which cNHEJ is not the preferable DSBs repair pathway. Besides, in yeasts like *S. cerevisiae*, it has been speculated that the absence of Ku-PK-Bind may have been compensated by alternate factors such as Mre11, Rad50, and Xrs2 (MRX) complexes [32].

It is noteworthy that some organisms exhibit variations in the domain architecture of Ku70 and Ku80, possibly reflecting the extent to which they rely on cNHEJ for DSBs repair. Moreover, the addition of a completely new domain, for instance, the APLF_PBZ domain in Ku70 of Amoebozoa, possibly enhances the strength of cNHEJ or functional diversity. The absence of certain domains in Ku70 or Ku80 may indicate the presence of alternative pathways for repairing DSBs. For example, unicellular yeast-like *S. cerevisiae* favors HR instead of NHEJ [33,34], which explains its incomplete Ku70 domain architecture.

Additionally, since cNHEJ is dispensable in parasitic eukaryotes like *Entamoeba, Trypanosoma,* and *Leishmania* species, the presence of the Ku70 paralog without the complete domain structure may be attributed to the loss of these domains in these organisms [25]. However, considering that the core domain is responsible for dimerization and DNA binding, the absence of additional domains may suggest alterations in the efficiency of the cNHEJ pathway. Lastly, the Ku protein present in *T. thermophila* transposon was found to harbor at least the core domain which may have resulted from horizontal gene transfer among the eukaryotes. While predicting the evolutionary trend solely based on the available domain structure remains challenging, it is evident that specific domains are consistently possessed and maintained by various organisms. Interestingly, even in cases where classical non-homologous end joining (cNHEJ) seems non-functional, certain domains are retained. A notable example is present in parasitic eukaryotes like *Trypanosoma cruzi*, which retain the Ku-PK-Bind domain despite favoring alternative pathways for NHEJ.

### A model of Ku inheritance

In the combined analysis of Ku70, Ku80, and prokaryotic Ku, distinct clades emerged, with Ku70 and Ku80 forming a closer relationship, separate from prokaryotic Ku. This separation implies a likely gene duplication of the Ku protein early in eukaryote evolution. Notably, in the evolutionary tree, eukaryotic Ku70 appears more closely related to archaeal Ku than to bacterial Ku, suggesting a possible inheritance of Ku from archaea to eukaryotes or a common ancestor for both, leading to the emergence of eukaryotic Ku in the earliest eukaryotic ancestor. Speciation events of the Ku protein might have occurred during early eukaryotic evolution after the possible vertical inheritance from prokaryotes to eukaryotes. It is difficult to reconstruct Ku gene evolution across such vast time scales and evolutionary divergent lineages. However, more comprehensive genomic knowledge and tools will enable us to grasp deeper understanding of early eukaryotic Ku protein evolution.

### Structural conservation and evolution of the Ku core protein across species

Structural analysis revealed that the beta-barrel motif within the core domain is highly conserved across the protein subsets. Our results align with previous studies, which demonstrate that the β-barrel ring domain is structurally and functionally conserved across species, despite divergences in the primary sequences of eukaryotic Ku70 and Ku80 subunits [35]. The maximum RMSD value observed was 5.871 when comparing the human Ku70 core with *S. cerevisiae* Ku70. This higher RMSD can primarily be attributed to the variation in the flexible loop regions that connect the conserved antiparallel beta sheets. Similarly, in the prokaryotic Ku core, the extra regions in the C-terminus, as compared to eukaryotic Ku, might contribute to higher RMSD values (Fig 6). Despite these variations in loop regions and additional sequences, the overall structure of the Ku core remains conserved, particularly the beta-barrel domain. This suggests that these domains have undergone minimal evolutionary changes, particularly in regions critical to their essential dimerization and DNA-binding function.

### Proposal for future experiments

While this study has offered us deeper insights into the evolutionary history of the Ku protein, it has raised multiple open-ended questions that could be addressed in the future. For instance, few parasitic eukaryotes contain only Ku70. Is it possible that there is a loss of Ku80 in eukaryotes with alternative DSB repair pathways? While Tadi. et. al has reported in-vitro homodimerization of Ku70, there remains an open-ended question of whether Ku70 can work independently in organisms devoid of Ku80 [36]. Moreover, some Ku protein sequences are annotated to have only the N-terminal Ku domain or SAP domain in the annotation databases, which makes the presence of intact Ku machinery a little blurry. While Ku proteins have been identified in early branching eukaryotes, more in-depth structural analysis will help us gain deeper insight into the Ku protein evolution along eukaryotic lineages.

## Materials and methods

### Sequence retrieval in prokaryotes

The Ku protein sequence of *Mycobacterium tuberculosis* [UniProt Accession ID: P9WNV3] was retrieved from UniProt. As Ku protein has been extensively studied in *M. tuberculosis,* its Ku beta-barrel domain sequence was used as a query to do protein blast in 122 chosen prokaryotes covering most of the families of bacteria. The non-redundant protein sequence database was used, and blastp was conducted using the default BLOSUM62 matrix, gap cost of Existence:11 Extension: 1, and conditional compositional score matrix adjustment. Only significant hits with an e-value less than 1e-5 and a percentage identity greater or equal to 30% were selected for multiple sequence alignment and phylogenetics.

### Sequence retrieval in Eukaryotes

To elucidate the diversity of Ku proteins within eukaryotes, we retrieved 1097 Ku70 sequences and 1256 Ku80 sequences found in eukaryotes from the OrthoDB database. OrthoDB is a user-friendly, well-sequenced, and annotated database of orthologous protein-coding genes across prokaryotes, eukaryotes, and viruses [37]. We used the ‘X-ray repair cross-complementing 6’ or Group ‘21093at2759’ group name for Ku70 and the ‘X-ray repair cross-complementing 5’ or Group ‘5884at2759’ group name for Ku80 protein. Once Fasta sequences were downloaded from orthoDB, conserved Ku domains in these sequences were identified using the NCBI Batch CD-Search Tool [38]. All sequences were trimmed to only include Ku70/Ku80 beta-barrel domains (CDD accession ID: pfam 02735). The trimmed sequences were further used for multiple sequence alignment and phylogenetic tree construction.

We selected 61 representative species covering most eukaryotic phyla to study the evolution of Ku protein across different domains of life (S2 Table). To remove database bias, we searched for Ku protein in OrthoDB and UniProt databases. We combined results from both database searches. Like the above, we isolated Ku70/Ku80 beta-barrel domain sequences for these species, which were further used for multiple sequence alignment and phylogenetic tree construction.

### Sequence retrieval in Archaea and viruses

We downloaded 19 archaeal sequences grouped under the ortholog group name ‘Archea protein Ku’ or Group ‘147616at2157’ from OrthoDB. Likewise, 11 viral Ku protein sequences grouped under the ortholog group name ‘Viral protein Ku’ or identifier number ‘5222at10239’ were also retrieved from OrthoDB. Like the eukaryotic sequences, we trimmed these Ku sequences only to retrieve Ku70/80 beta-barrel domains, which were further used for multiple sequence alignment and phylogenetic tree construction.

### Domain specification

Ku protein FASTA sequences retrieved from databases were provided as input to InterPro for domain identification [39]. Various Ku70 and Ku80 domain architectures of the respective species were drawn using Adobe Illustrator 2023 (Fig 2 and 3).

### Multiple sequence alignment and phylogenetic tree construction

We constructed three maximum likelihood trees to study the evolutionary history of Ku proteins across bacteria, archaea, and eukaryotes. For each tree, multiple sequence alignment was generated using the Muscle Algorithm in Seaview software using default settings [31,40]. The resulting multiple alignment was used to construct a phylogenetic tree using PhyML [41]. Automatic model selection based on the lowest BIC (Bayesian Information Criterion) was done using Smart Model Selection (SMS) in PhyML [42]. BioNJ starting tree construction was selected for optimization. The support for each branch was established using Shimodaira–Hasegawa [SH]-aLRT (appromixate Likelihood Ratio Test) [41]. Phylogenetic trees were rooted using MEGA11: Molecular Evolutionary Genetics Analysis version 11 and nodes with support values of less than 50% were condensed [43].

### Structure models of Ku core proteins

The structural models of a subset of Ku core proteins from different species were predicted using the AlphaFold server [44]. From the generated models, the highest-confidence structures were selected for further analysis. These selected models were then visualized and analyzed using PyMOL3.1.3(Jumper et al., 2021; PyMOL User’s Guide, 2004)(Jumper et al., 2021; PyMOL User’s Guide, 2004)(Jumper et al., 2021; PyMOL User’s Guide, 2004) to assess their topology and secondary structural elements. For comparison, the core domains of the Homo sapiens Ku70 and Ku80 proteins were extracted from the experimentally solved Ku70/80 heterodimers bound to DNA (PDB: 1JEY). The AlphaFold-predicted protein structures were superimposed onto these experimental models, and root mean square deviation (RMSD) values were calculated to assess the structural similarity between the predicted and experimental structures.

## Supporting information

S1 Fig Phylogenetic tree of 1097 Ku70 protein sequences retrieved from OrthoDB. Ku70 sequences were trimmed to obtain Ku70/80 beta-barrel sequences. 1097 sequences obtained were inferred by using the Maximum Likelihood method. Phylogenetic trees were drawn using PhyML. Automatic model selection based on the lowest BIC (Bayesian Information Criterion) was done using Smart Model Selection (SMS) in PhyML. Support for each branch was established using Shimodaira–Hasegawa [SH]-aLRT (approximate Likelihood Ratio Test). Nodes with support values of less than 50% were condensed using Mega-11 and the tree was annotated using FigTree and Abode Illustrator 2023.
(PDF)

S2 Fig Phylogenetic tree of 1256 Ku80 protein sequences retrieved from OrthoDB. Ku80 sequences were trimmed to obtain Ku70/80 beta-barrel sequences. 1256 sequences obtained were inferred by using the Maximum Likelihood method. Phylogenetic trees were drawn using PhyML. Automatic model selection based on the lowest BIC (Bayesian Information Criterion) was done using Smart Model Selection (SMS) in PhyML. Support for each branch was established using Shimodaira–Hasegawa [SH]-aLRT (approximate Likelihood Ratio Test). Nodes with support values of less than 50% were condensed using Mega-11 and the tree was annotated using FigTree and Abode Illustrator 2023.
(PDF)

S3 Fig Predicted Ku core protein structures from different species using AlphaFold. The best AlphaFold models for the Ku core proteins from various species were selected and visualized. The structures of the predicted proteins are shown to highlight the core domain’s antiparallel beta-barrel, which is highly conserved across species.
(TIF)

S1 Table RMSD values for the superimposition of the experimental human Ku core (Ku70 and Ku80) with the predicted core domains of Ku proteins from various species. (DOCX)

S2 Table UniprotKB entry ID for representative eukaryotic species harboring Ku protein Uniprot Entry ID for (a) Ku70 protein sequences retrieved for constructing Fig 2a. (b) Ku80 protein sequences retrieved for constructing Fig 2b.
(DOCX)

## Abbreviations

DSBs: Double-stranded Breaks
NHEJ: Non-homologous End Joining
HR: Homologous recombination
DNA-PK: DNA-dependent protein kinase
PIKK: Phosphoinositide 3- kinase-related kinase
vWA: von Willebrand A
CTD: C-terminal Domain
NCBI: National Centre for Biotechnology Infromation; UniProt, Universal Protein Resource
BLAST: Basic Local Alignment Search Tool
BLASTP: Protein-protein BLAST.
