# Supplementary figures and images for: Evolutionary history of the DNA repair protein, Ku, in eukaryotes and prokaryotes
Source: PLoS One. 2025 Mar 25;20(3):e0308593. doi: 10.1371/journal.pone.0308593 (PMC11936186; doi:10.1371/journal.pone.0308593)

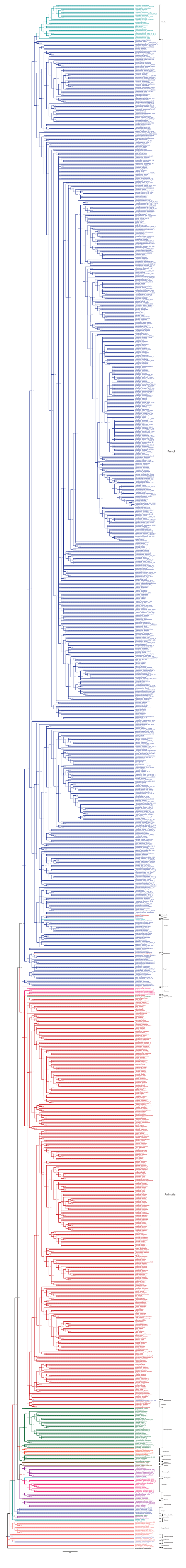

Supplement: S1 Fig — Ku70 sequences were trimmed to obtain Ku70/80 beta-barrel sequences. 1097 sequences obtained were inferred by using the Maximum Likelihood method. Phylogenetic trees were drawn using PhyML. Automatic model selection based on the lowest BIC (Bayesian Information Criterion) was done using Smart Model Selection (SMS) in PhyML. Support for each branch was established using Shimodaira–Hasegawa [SH]-aLRT (approximate Likelihood Ratio Test). Nodes with support values of less than 50% were condensed using Mega-11 and the tree was annotated using FigTree and Abode Illustrator 2023. (PDF) [file pone.0308593.s001.pdf]

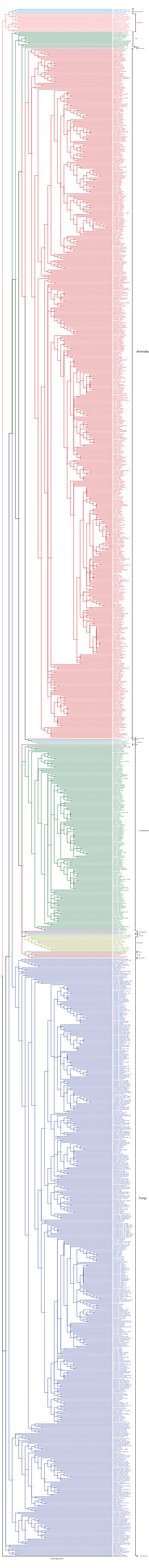

Supplement: S2 Fig — Ku80 sequences were trimmed to obtain Ku70/80 beta-barrel sequences. 1256 sequences obtained were inferred by using the Maximum Likelihood method. Phylogenetic trees were drawn using PhyML. Automatic model selection based on the lowest BIC (Bayesian Information Criterion) was done using Smart Model Selection (SMS) in PhyML. Support for each branch was established using Shimodaira–Hasegawa [SH]-aLRT (approximate Likelihood Ratio Test). Nodes with support values of less than 50% were condensed using Mega-11 and the tree was annotated using FigTree and Abode Illustrator 2023. (PDF) [file pone.0308593.s002.pdf]

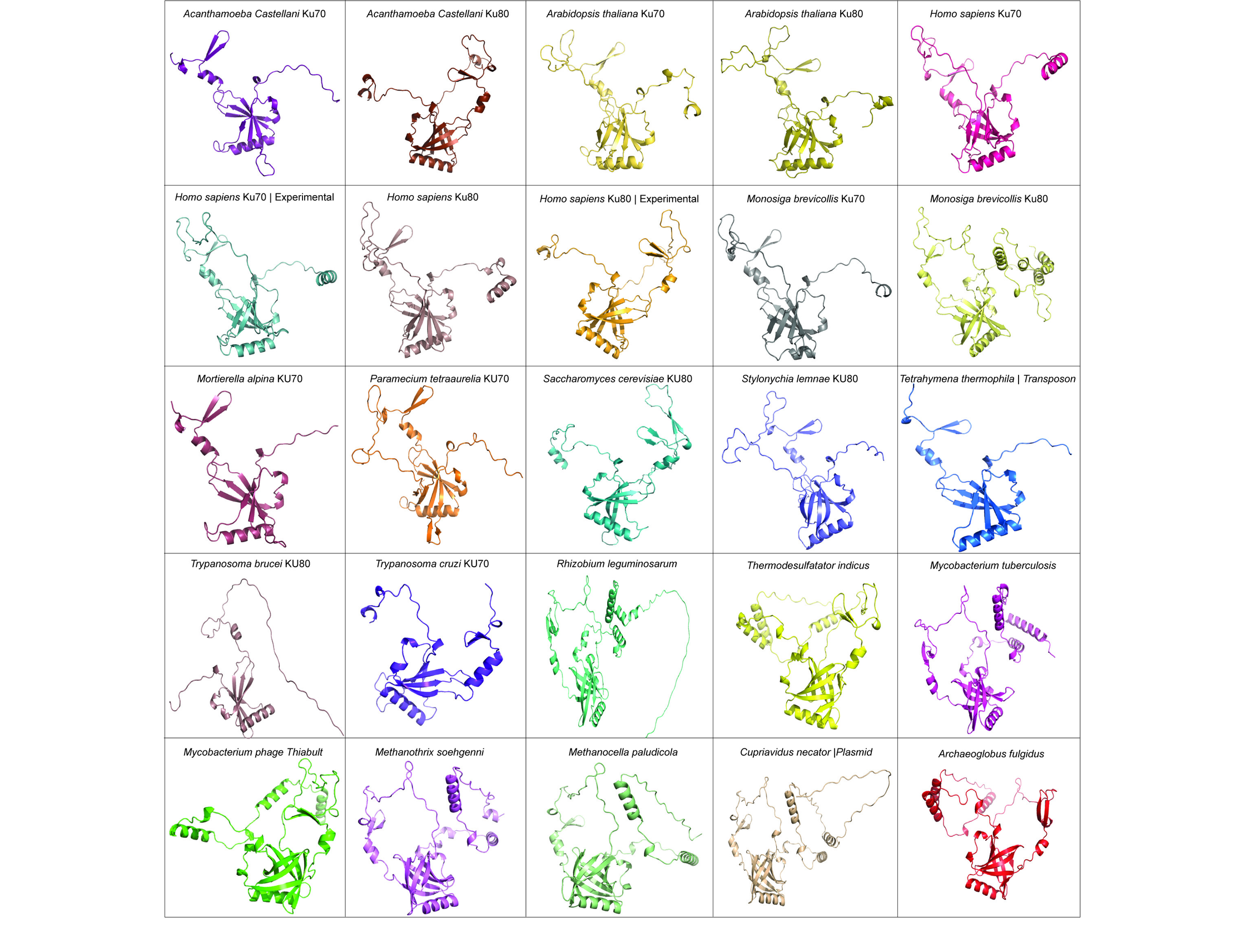

Supplement: S3 Fig — The best AlphaFold models for the Ku core proteins from various species were selected and visualized. The structures of the predicted proteins are shown to highlight the core domain’s antiparallel beta-barrel, which is highly conserved across species. (TIF) [file pone.0308593.s003.tif]
