# Supplementary material for: Evolutionary history of the DNA repair protein, Ku, in eukaryotes and prokaryotes
Source: PLoS One. 2025 Mar 25;20(3):e0308593. doi: 10.1371/journal.pone.0308593 (PMC11936186; doi:10.1371/journal.pone.0308593)
Supplement: S1 Table — (DOCX) [file pone.0308593.s004.docx]

| **Species** | *Homo sapiens*\|KU70 (Experimental) | *Homo sapiens*\|KU80 (Experimental) |
| --- | --- | --- |
| *Mycobacterium phage Thibault* | 3.965 | 2.701 |
| *Mycobacterium tuberculosis* | 3.499 | 2.954 |
| *Thermodesulfatator indicus* | 3.091 | 2.636 |
| *Archaeoglobus fulgidus* DSM 430 | 2.672 | 3.2 |
| *Nitrososphaera sp* AFS | 3.984 | 3.995 |
| *Methanothrix soehngenii* GP6 | 3.308 | 3.397 |
| *Opitutus terrae* | 2.801 | 3.263 |
| *Rhizobium leguminosarum* | 3.042 | 4.056 |
| *Cupriavidus necator*\|P\| | 3.573 | 2.861 |
| *Methanocella paludicola* SANAE | 3.047 | 2.715 |
| *Saccharomyces cerevisiae*\|KU80 | 5.871 | 1.957 |
| *Trypanosoma cruzi* 3\|KU70 | 1.553 | 2.063 |
| *Mortierella alpina*\|KU70 | 0.724 | 1.923 |
| *Paramecium tetraurelia*\|KU70 | 0.71 | 3.212 |
| *Acanthamoeba castellanii*\|KU70 | 0.677 | 1.811 |
| *Homo sapiens*\|KU70 | 1.557 | 2.551 |
| *Monosiga brevicollis*\|KU70 | 0.713 | 2.849 |
| *Arabidopsis thaliana*\|KU70 | 1.122 | 3.519 |
| *Puccinia graminis*\|KU80 | 2.364 | 2.175 |
| *Arabidopsis thaliana*\|KU80 | 2.493 | 1.314 |
| *Acanthamoeba castellanii*\|KU80 | 2.141 | 0.91 |
| *Monosiga brevicollis*\|KU80 | 2.533 | 1.488 |
| *Homo sapiens*\|KU80 | 2.442 | 1.785 |
| *Stylonychia lemnae*\|KU80 | 3.2 | 1.318 |
| *Tetrahymena thermophila*\|T\| | 1.989 | 0.7 |
| *Trypanosoma brucei*\|KU80 | 1.152 | 1.078 |
