# Supplementary material for: Evolutionary history of the DNA repair protein, Ku, in eukaryotes and prokaryotes
Source: PLoS One. 2025 Mar 25;20(3):e0308593. doi: 10.1371/journal.pone.0308593 (PMC11936186; doi:10.1371/journal.pone.0308593)
Supplement: S2 Table — (b) Ku80 protein sequences retrieved for constructing Fig 2b. (DOCX) [file pone.0308593.s005.docx]

**a.**

| **Organism** | **UniProt Entry** |
| --- | --- |
| **Animalia** | |
| *Hydra vulgaris* | T2MGH8 |
| *Monodelphis domestica* | A0A5F8GS76 |
| *Branchiostoma floridae* | C3Z1F8 |
| *Strongylocentrotus purpurat* | A0A7M7P5E8 |
| *Nematostella vectensis* | A7RH48 |
| *Homo sapiens* | P12956 |
| **Choanoflagellate** | |
| *Monosiga brevicollis* | A9UYE6 |
| **Fungi** | |
| *Malassezia globosa* | A8PYA1 |
| *Candida albicans* | A0A1D8PF81 |
| *Aspergillus oryzae* | Q2MHH4 |
| *Neurospora crassa* | A0A0B0DTB4 |
| *Mortierella alpina* | A0A9P6M642 |
| *Ustilago maydis* | A0A0D1E1V7 |
| *Laccaria bicolor* | B0D3W2 |
| *Puccinia graminis_1* | A0A5B0PY30 |
| *Puccinia graminis_2* | A0A5B0QNB7 |
| *Schizosaccharomyces pombe* | O94395 |
| *Talaromyces marneffei* | PMAA_037760 |
| *Saccharomyces cerevisiae* | P32807 |
| **Ichthyospora** | |
| *Sphaeroforma arctica* | A0A0L0FM70 |
| **Amoebozoa** | |
| *Entamoeba invadens* | A0A0A1TW30 |
| *Entamoeba histolytica* | A0A5K1VDL8 |
| *Acanthamoeba castellanii* | L8HDL0 |
| *Dictyostelium discoideum* | Q54MA9 |
| **Alveolata** | |
| *Oxytricha trifallax* | J9F1X1 |
| *Stylonychia lemnae* | A0A078AP83 |
| *Tetrahymena thermophila_1* | A4VEG9 |
| *Tetrahymena thermophila_2* | I7MAM7 |
| *Tetrahymena thermophila_T* | A0A1B9C248 |
| *Paramecium tetraurelia_1* | Q4LBE9 |
| *Paramecium tetraurelia_2* | Q4LBE8 |
| **Chloroplastida** | |
| *Chlamydomonas reinhardtii* | A0A2K3D1K3 |
| *Ostreococcus lucimarinus* | A0A7R9T1F6 |
| *Arabidopsis thaliana* | Q9FQ08 |
| *Volvox carteri* | D8UMJ0 |
| *Zea Mays* | A0A3L6FTT5 |
| **Discoba** | |
| *Trypanosoma cruzi_1* | A0A7J6XZ19 |
| *Trypanosoma cruzi_2* | A0A2V2WYV1 |
| *Trypanosoma cruzi_3* | A0A2V2XDX7 |
| *Trypanosoma cruzi_4* | Q4CY05 |
| *Trypanosoma brucei* | Q95PL9 |
| *Leishmania infantum* | A0A6L0XIU6 |
| *Leishmania major* | E9ADW3 |
| *Leishmania donovani* | A0A504XU21 |

**b.**

| **Organism** | **UniProt Entry** |
| --- | --- |
| **Animalia** | |
| *Hydra vulgaris* | T2MHH9 |
| *Monodelphis domestica* | A0A5F8G636 |
| *Branchiostoma floridae* | A0A9J7MMM3 |
| *Strongylocentrotus purpurat_1* | A0A7M7RCH6 |
| *Strongylocentrotus purpurat_2* | A0A7M7SZY5 |
| *Nematostella vectensis* | A7SI21 |
| *Homo sapiens* | P13010 |
| **Choanoflagellate** | |
| *Monosiga brevicollis* | A9VC10 |
| **Fungi** | |
| *Malassezia globosa* | A8QAH0 |
| *Candida albicans* | A0A1D8PSH5 |
| *Aspergillus oryzae* | Q2MHH2 |
| *Neurospora crassa* | Q7RX73 |
| *Mortierella alpina* | A0A9P6J8Q6 |
| *Ustilago maydis* | A0A0D1DRI5 |
| *Laccaria bicolor* | B0CPI8 |
| *Puccinia graminis* | A0A5B0P965 |
| *Schizosaccharomyces pombe* | Q9HGM8 |
| *Talaromyces marneffei* | A0A7C8KXY9 |
| *Saccharomyces cerevisiae* | Q04437 |
| **Ichthyospora** | |
| *Sphaeroforma arctica* | A0A0L0FRU7 |
| **Amoebozoa** | |
| *Acanthamoeba castellanii* | L8H854 |
| *Dictyostelium discoideum* | Q54LY5 |
| **Alveolata** | |
| *Oxytricha trifallax* | J9HU40 |
| *Stylonychia lemnae* | A0A078A0N2 |
| *Tetrahymena thermophila* | I7LWU7 |
| *Paramecium tetraurelia* | A0BLP5 |
| **Chloroplastida** | |
| *Chlamydomonas reinhardtii* | A8ICD8 |
| *Ostreococcus lucimarinus* | A4S0U7 |
| *Arabidopsis thaliana* | Q9FQ09 |
| *Volvox carteri* | D8U689 |
| *Zea Mays* | B6SWG7 |
| **Discoba** | |
| *Trypanosoma cruzi* | V5BGZ6 |
| *Trypanosoma brucei* | Q585N5 |
| *Leishmania infantum* | A0A6L0XLG9 |
| *Leishmania major* | Q4Q7S5 |
| *Leishmania donovani* | A0A3Q8IRQ2 |
